# Revealing cellular and molecular complexity of the central nervous system using single cell sequencing

**DOI:** 10.1186/s13287-018-0985-z

**Published:** 2018-09-13

**Authors:** Zhiwei Zeng, Nan Miao, Tao Sun

**Affiliations:** 1 Center for Precision Medicine, School of Medicine and School of Biomedical Sciences, Huaqiao University, Xiamen, Fujian, 361021 China; 2 Department of Cell and Developmental Biology, Cornell University Weill Medical College, 1300 York Avenue, Box 60, New York, NY 10065 USA

## Abstract

The mammalian central nervous system (CNS) is one of the most complex systems, with thousands of cell types and subtypes with distinct and unique morphology and gene expression profiles. Based on classic histological methods and conventional cellular and molecular approaches, single cell sequencing is becoming a powerful tool to uncover the complexity of the CNS. In this review, we summarize the principle of single cell sequencing and highlight its use for studying the development of neural stem cells, neural progenitors, and distinct neurons. By revealing transcriptomes in each individual cell using single cell sequencing, we are now able to dissect the cellular heterogeneity of a hundred billion cells in the CNS and comprehensively investigate mechanisms of brain development and function at the cellular and molecular levels.

## Background

Advancing our knowledge in neuroscience relies on the fast development of modern technologies, such as next-generation sequencing (NGS), optogenetic modulation, and CRISPR-Cas9 [1–3]. These technologies have been used to investigate brain development and function, for example, brain morphology and electrophysiology. Recently, single cell sequencing has explored new aspects of stem cell biology and neuroscience and generated exciting discoveries based on traditional classification of cell types and subtypes in the central nervous system (CNS).

In this review, we summarize the principle of single cell sequencing and highlight its application in neuroscience. We first introduce methods of single cell sequencing, such as single cell isolation, whole-genome amplification (WGA), and whole-transcriptome amplification (WTA). We next reveal the application of single cell sequencing for classifying cell types in the CNS, for understanding molecular mechanisms of development of neural stem cells and neural progenitors in human brains, and for modeling human brain formation and disorders.

### The principle of single cell sequencing

**Fig. 1:**
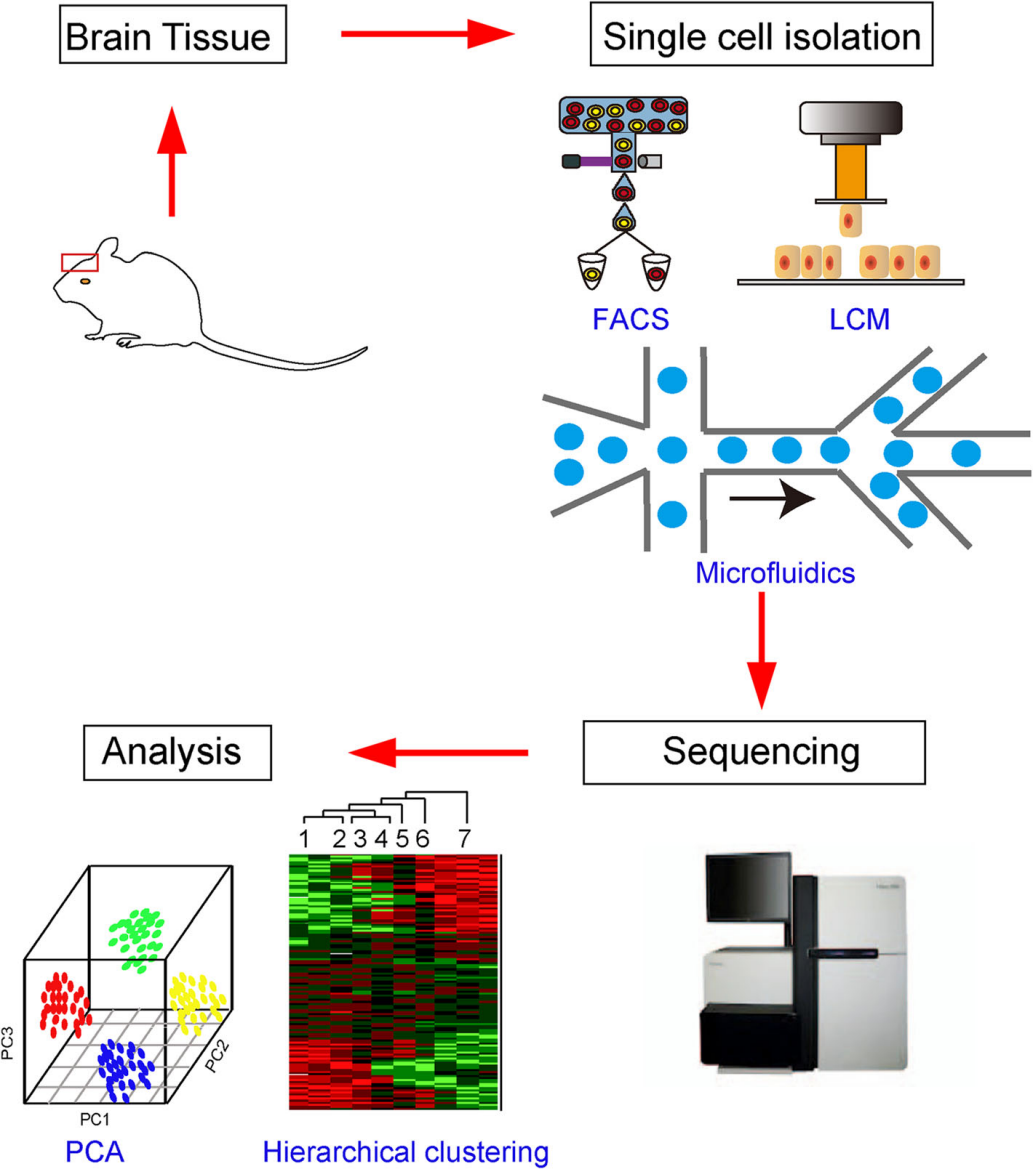
Single cell sequencing flow chart. Brain tissues from the brain region of interest are collected, then single cells are captured by fluorescence-activated cell sorting (*FACS*), laser capture microdissection (*LCM*), and microfluidics (Fluidigm C1 and 10× Genomics Chromium). Once single cells are captured, DNA or RNA are amplified for the next sequencing step. Principle component analysis (*PCA*) and hierarchical clustering are ideal tools to analyze the single cell sequencing data and to classify cell types

The general procedure of single cell sequencing consists of six steps: isolation of single cells; cell lysis to obtain DNA or RNA; addition of barcodes in single cells; amplification of DNA and RNA for sequencing; library preparation and sequencing; and data analysis (Fig. 1). Hierarchical clustering and principle component analysis (PCA) have been used to verify novel cell populations and distinct cell types through identification of new markers in the single cell transcriptomes.

#### Single cell isolation

Single cell isolation is the first key step of single cell sequencing. Several useful methods are available to capture single cells from tissues or cell cultures, such as fluorescence-activated cell sorting (FACS), laser capture microdissection (LCM), manual cell picking, microfluidic, and the use of microwells.

FACS is a classic method to isolate individual cells from brain tissue and cultured cells based on their expression of a reporter gene or their antigens against specific antibodies [4, 5] (Fig. 1). Because FACS requires a large number of cells in suspension as starting material, it is not suitable to capture low-abundance cell subpopulations.

The LCM method can capture cells under direct microscopic visualization, which permits rapid one-step sectioning of complex, heterogeneous tissue to achieve spatial information [6] (Fig. 1).

**Fig. 2:**
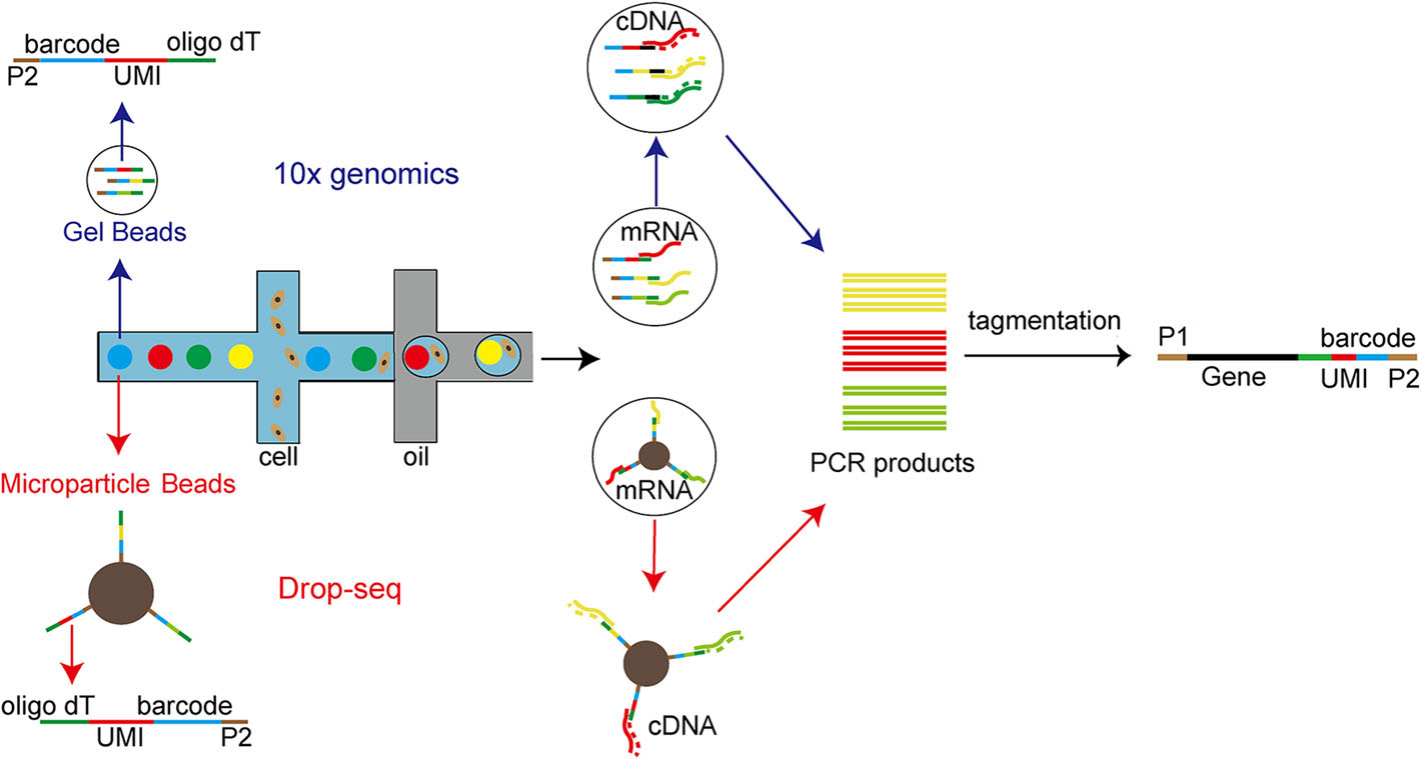
Flow chart of droplet-based single cell RNA sequencing. In the Drop-seq, one channel contains single cells for analysis and the other contains microparticle beads. The surface of a microparticle bead binds oligonucleotides that consist of oligo dT (*green*), a unique molecular identifier (UMI; *red*), a cell barcode (*blue*), and a PCR primer (*brown*). Immediately after droplet formation, cells are lysed and mRNAs released and then hybridized with oligonucleotides on the surface of the microparticle beads based on oligo dT binding. Droplets are then broken and mRNAs are reverse-transcribed in bulk and amplified for sequencing using PCR. Moreover, in the 10× Genomics platform, one channel contains single cells for analysis and the other contains gel beads mixed with oligonucleotides that consist of oligo dT, UMI, cell barcode, and a PCR primer. Cells and reagents are next mixed with gel beads. After cell lysis, their mRNAs are released and hybridized with oligonucleotides based on oligo dT binding, and are next reverse-transcribed in bulk and amplified for sequencing using PCR. *P1* and *P2* are PCR primers for establishing libraries for Illumina sequencing

Microfluidics is a newly developed, highly integrated system that allows sequential processing of small volumes of fluids in channels with dimensions of tens to hundreds of micrometers to achieve single cell culture and sequencing [7]. Several microfluidics platform are available, such as the Fluidigm C1, Drop-seq, and 10× Genomics Chromium. The procedure of the Fluidigm C1 platform includes cell capture, verification, cell lysis, reverse transcription and pre-amplification. Because the capture sizes are tuned to 5–10, 10–17, and17–25 μm in diameter, this system is suitable only for cells that are relatively homogeneous in size. The low capture efficiency also limits its use in capturing and sequencing sticky or non-spherical cells [8]. The Drop-seq and 10× Genomics Chromium systems are droplet-based techniques that enable processing of tens of thousands of cells in a single experiment (Fig. 2). The Drop-seq device allows co-flow of two aqueous solutions across an oil channel: one flow contains microparticle beads that bind barcoded oligonucleotides and the other contains single cells for analysis (Fig. 2) [9]. In the 10× Genomics Chromium system, cells combined with reverse transcription reagents in one channel and gel beads loaded with barcoded oligonucleotides from another channel are mixed to form Gel bead in Emulsion (GEM) (Fig. 2) [10]. After droplet formation, cells are lysed and mRNAs released, which are then hybridized with oligonucleotides based on oligo dT. Droplets are then broken and mRNAs reverse-transcribed in bulk and amplified for sequencing using PCR (Fig. 2).

In microwell sequencing, individual cells are trapped in an agarose microarray and mRNAs subsequently captured on magnetic beads for sequencing [11]. In addition, split-pool ligation-based transcriptome sequencing (SPLiT-seq) eliminates the need to separate individual cells by adding different barcodes to cells over several rounds, so each cell has a unique combination of barcodes for sequencing [12].

#### Adding barcodes in single cells

**Fig. 3:**
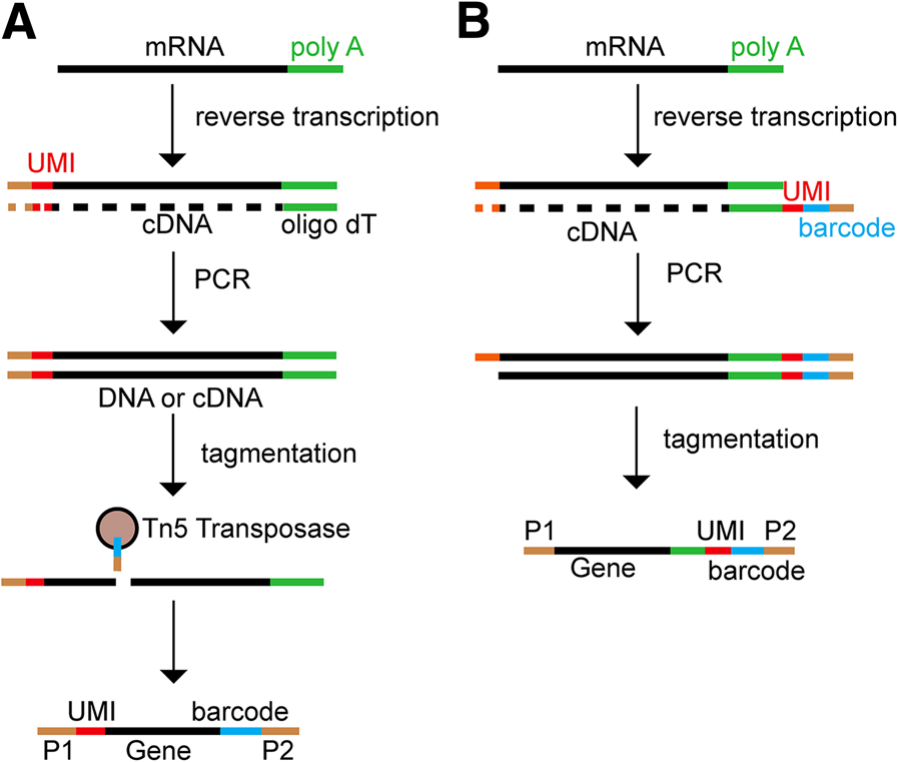
Two methods to add barcode in a single cell. **a** cDNA is reverse-transcribed and amplified using the oligo dT primer (*green*) in the presence of a template-switching oligonucleotide that contains a unique molecular identifier (*UMI*; *red*) and a PCR primer for Illumina sequencing (*brown*). After amplification, tagmentation is performed using Tn5 transposase carrying a specific cell barcode (*blue*) and a PCR primer. **b** cDNA is reverse-transcribed and amplified using an oligonucleotide that consists of oligo dT, UMI, a cell barcode, and a PCR primer, and then tagmented after amplification. *P1* and *P2* are PCR primers for establishing libraries for Illumina sequencing

Two strategies are most frequently used to add barcodes in single cells in order to distinguish individual cells (Fig. 3). One method is to use Tn5 transposase carrying a specific barcode to add a barcode after amplification of cDNA using oligo dT and unique molecular identifiers (UMI) (Fig. 3a). Another method is to design a primer containing an oligo dT, barcode, and PCR primer which adds a cell-unique barcode when the first cDNA strand is synthesized (Fig. 3b). Once a barcode is added, DNA and cDNA in a single cell are ready for amplification.

#### Single cell DNA sequencing

To meet the demands of next-generation sequencing, the amount of DNA in a single cell (approximately 6 pg) needs to be amplified using whole-genome amplification (WGA) [13]. Three methods have been applied in WGA: degenerate oligonucleotide-primed PCR (DOP-PCR), multiple displacement amplification (MDA), and multiple annealing and looping-based amplification cycles (MALBAC).

**Fig. 4:**
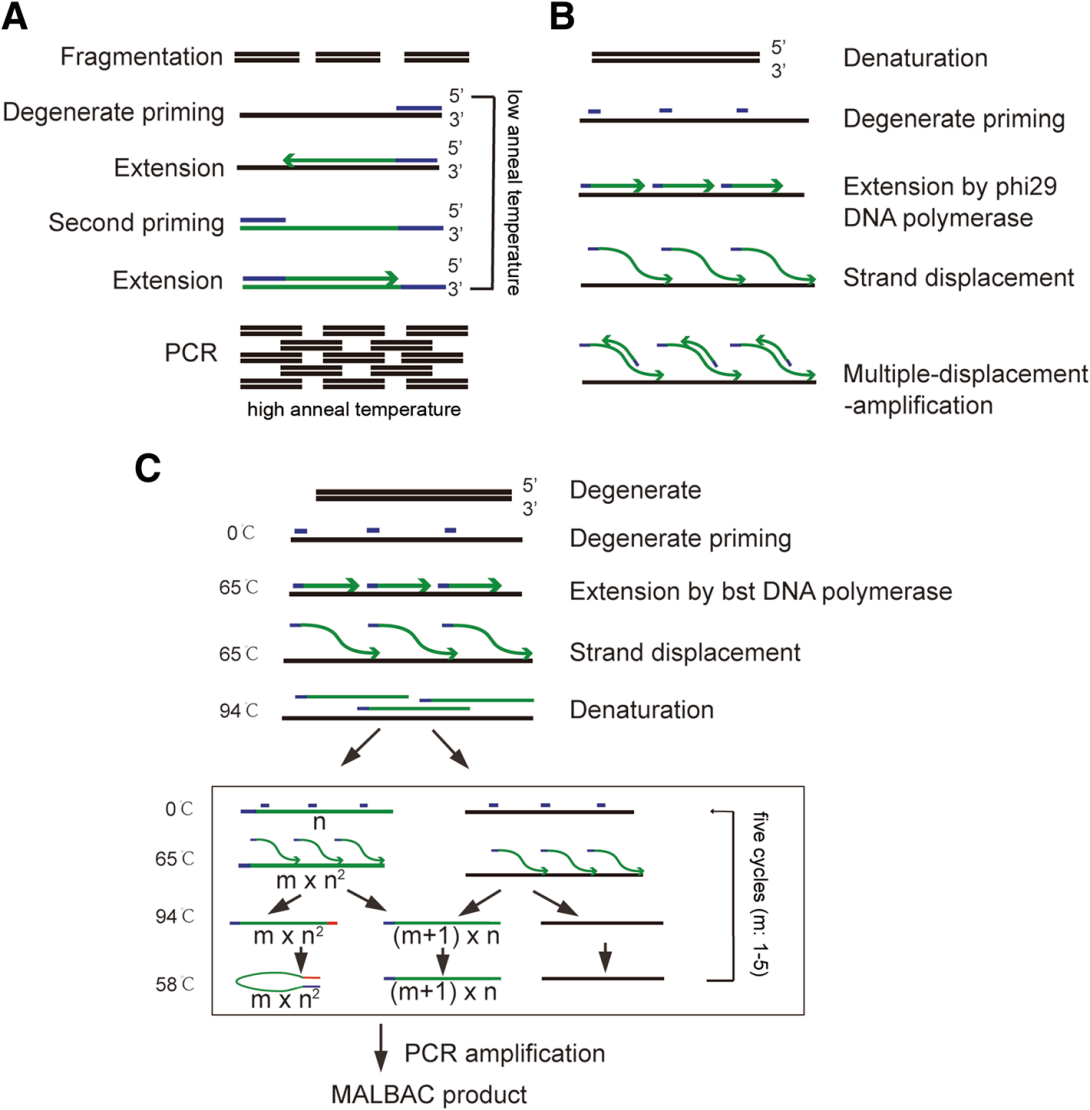
Whole-genome amplification methods for single cell sequencing. **a** Degenerate oligonucleotide-primed PCR (DOP-PCR). The 3′ end of the degenerate oligonucleotide primer (the random six nucleotides) are annealed to the genomic template, allowing the primer to initiate PCR, and PCR fragments are generated to contain the full length of the oligonucleotide primer at one end and the complementary sequence at the other end. Subsequently, the temperature is increased to amplify the DNA fragments. **b** Multiple displacement amplification (MDA). Double-stranded DNA are melted and random primers are bound to the DNA strand. Branched structures are produced under isothermal condition using the phi29 DNA polymerase. **c** Multiple annealing and looping-based amplification cycles (MALBAC). Double-stranded DNA is denatured into single strands at 94 °C, then single-stranded DNA templates are homogeneously bound with random primers at 0 °C. Semi-amplified products (n) are further amplified to produce full amplicons during a subsequent five temperature cycles (m), and the complete amplification products are 5′ and 3′ complementary to each other. Cyclization of the complete amplification product is performed with the temperature dropped to 58 °C to prevent further amplification and hybridization of the sequence. Semi-amplification products and genomic DNA continue to circulate to generate the complete amplification product

DOP-PCR is widely used in WGA. This method first amplifies the DNA template using a low annealing degenerate primer extension on the DNA template and then amplifies the previous products at a high annealing temperature [14] (Fig. 4a). Because the characteristics of PCR magnify the diversity of different sequences in the genome, DOP-PCR has a low physical coverage of the genome (approximately 10%). This method can accurately retain copy number levels, which makes it an ideal method to detect single cell copy-number variants (CNVs) [15, 16].

MDA is a useful method for identification of SNPs, having a high physical coverage of a single cell genome (approximately 90%) due to the use of special DNA polymerase. For instance, Phi29 DNA polymerase is isolated from *Bacillus subtilis* and Bst DNA polymerase is isolated from *Bacillus stearothermophilus* [17–19]. Phi29 DNA polymerase is considered a better choice for MDA because it shows higher efficiency and affinity and lower error rates than Bst DNA polymerase [20]. MDA produces branched structures under the isothermal conditions through extending random primers (Fig. 4b). Because it may lead to uneven genome coverage, it is not suitable for CNV validation [19].

MALBAC uses PCR amplification of circular DNA fragments with Bst DNA polymerase [21] (Fig. 4c). MALBAC is used only to make copies of the original genomic DNA by protecting the amplification products, in which the 3′ end is complementary to the sequence on the 5′ end in full amplicons [16]. The two ends hybridize to form looped DNA, which can efficiently prevent the full amplicon from being used as a template, therefore enabling close to linear amplification [21]. Thus, MALBAC not only has accuracy for CNV detection, but also features a low false negative rate for SNV detection.

All these methods provide exponential amplification, which creates amplification bias and errors. A new WGA method, linear amplification via transposon insertion (LIANTI), can reduce exponential amplification bias and errors by combining Tn5 transposition and T7 in vitro transcription [22].

Moreover, single nucleus exome sequencing (SNES) is a newly developed method to capture single cells in G1/0 or G2/M phase in a cell cycle. After amplification of single cell genomes using MDA, SNES can reduce technical errors during the single cell sequencing of exomes and genomes [23, 24].

#### Single cell RNA sequencing

Due to the tiny amount of total RNA (approximately 10 pg) and mRNA (approximately 0.1 pg) in a single cell [25], to perform single cell RNA sequencing, RNA must be amplified by whole transcriptome amplification (WTA) using either the oligo dT-anchor approach (Tang2009 method) [26–30], template-switching approach (SMART-seq/SMART-seq2 method) [9, 31–37], in vitro transcription (IVT)-based linear amplification (CEL-seq/CEL-seq2 method) [38–41], or designed primer-based PCR [42, 43].

**Fig. 5:**
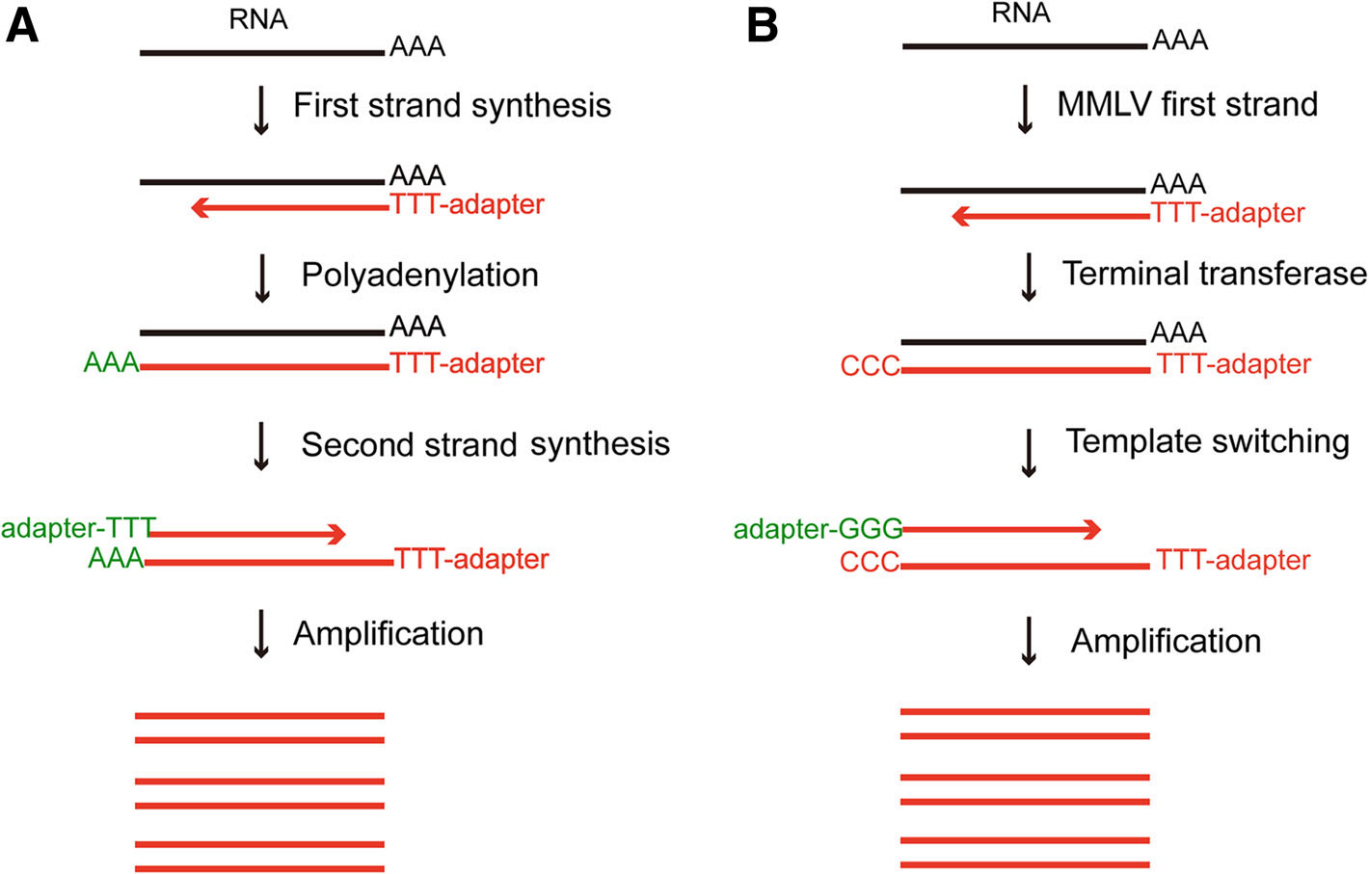
Whole transcriptome amplification methods for single cell sequencing. **a** Oligo dT-anchor approach. mRNA is reverse-transcribed with an oligo-dT anchored primer, and poly(A) is added to the 3′ end of the first-strand cDNA to synthesize the second strand cDNA. **b** Template-switching approach. mRNA is reverse-transcribed by Moloney murine leukemia virus (MMLV) reverse transcriptase, and a few non-templated C nucleotides are added to the 3′ end of the first-strand cDNA to synthesize the second strand cDNA

In the Tang method, mRNAs are reverse-transcribed into cDNAs using a poly(T) primer with an anchor sequence, then poly(A) tails are added to the first strand cDNAs at the 3′ end. The second-strand cDNAs are synthesized using poly(T) primers with another anchor sequence [25, 26] (Fig. 5a). The drawback of this method is that the limited length of the reverse-transcription product causes a strong 3′ mRNA bias [44].

The SMART-seq method can mitigate this bias by adding a new non-templated C nucleotide to the 5′ end of cDNA, which uses Moloney murine leukemia virus (MMLV) reverse transcriptase to amplify the full-length cDNA [33] (Fig. 5b). SMART-seq2 can obtain increased cDNA yields from single cells and also has higher sensitivity, fewer technical biases, and less variability by more refined reverse transcription, template switching, and pre-amplification [34]. However, exponential amplification may lead to gene expression profiles represented by shorter and less enriched G-C-bias amplicons [45].

The CEL-seq method can overcome this problem to achieve linear amplification. By using a primer designed with an anchored poly(T), a unique barcode, the 5′ Illumina sequencing adaptor, and a T7 promoter, the second-strand synthesis is performed and then the cDNA samples are pooled and the IVT reaction performed [38]. RNAs are then fragmented and ligated with an Illumina 3′ adaptor and reverse transcribed to DNA for sequencing. In the CEL-seq2 method, the sensitivity of the CEL-seq is improved by performing another round of reverse transcription with random primers [39]. Additionally, the UMI labeling technique is applied in single cell RNA sequencing, which can increase the efficiency of cDNA synthesis and quantitative accuracy [46].

### Application of single cell sequencing in the nervous system

Single cell sequencing has changed our fundamental understanding of tumor heterogeneity, clonal evolution, and metastatic dissemination [23, 47–52]. It has also been used to study the immune system, to define immune cell subpopulations, and to examine gene expression variability, splice variants, and gene-regulatory networks [53–55]. In addition to the study of tumors and immunology, neuroscience is another field where single cell sequencing technology has a great advantage.

#### Classification of cell types in the CNS

The classification of cell types in the CNS has challenged the greatest minds in the history of neuroscience. Early neuroscientists used histological knowledge (morphological characteristics and localization) to classify cell types in the CNS. Ramón y Cajal, the pioneer in neuroscience, systematically described cellular structures in the brain using the Golgi stain method, which has guided neuroscience studies for more than a century [56]. Afterwards, cellular biology approaches have been used to label specific cell surface and nuclear antigens with antibodies to distinguish cell types, and then DNA and RNA sequencing has been used to profile differential gene expression from bulk tissues or cultured cells. However, few known cell surface and nuclear protein markers for specific cells and limited distinction of cell morphology have restricted comprehensive understanding of cellular complexity in the CNS [57]. Single cell sequencing has allowed characterization of various cell types in the CNS at the molecular level (Table 1).

**Table 1:** Summary of cell types in the CNS identified by single cell sequencing

Region in the CNS	Cell number	Major finding	Reference
Mouse somatosensory and hippocampal CA1	3,005	7 pyramidal neuron, 16 interneuron, 6 oligodendrocyte subclasses	[59]
Mouse primary visual cortex	1,679	23 GABAergic interneuron and 19 glutamatergic neuron subtypes	[60]
Mouse neocortex layer 1	58	Elongated neurogliaform and bouquet cell types	[31]
Postmortem human brain	3,227	8 excitatory and 8 inhibitory neuronal subtypes	[62]
Postmortem human brain	35,289	35 subpopulations of neuronal and non-neuronal cell types	[63]
Mouse retina	44,808	20 amacrine and 8 bipolar cell types	[9]
Mouse retina	25,000	15 types and 2 novel types of bipolar cells	[65]
Mouse hypothalamus	3,131	32 glutamatergic neurons and 13 GABAergic neurons	[68]
Mouse hypothalamus	3,319	15 glutamatergic neurons and 18 GABAergic neurons	[69]
Mouse olfactory	178	Immature olfactory sensory neurons express multiple olfactory receptor genes	[71]
Mouse olfactory	78	Immature olfactory sensory neurons express low levels of multiple olfactory receptor genes	[72]

#### Cerebral cortex

The cerebral cortex is a central brain region that controls complex cognitive behaviors, such as sensorimotor integration, memory, and social behaviors [58]. Recently, single cell RNA sequencing has been used to identify different neuronal types and subtypes and to discover novel cell-specific markers in the mouse cortex [31, 59, 60]. One study analyzed 3,005 cells isolated from the mouse somatosensory cortex and hippocampal CA1 [59]. These 3,005 cells have been classified into nine molecularly distinct classes by clustering cells such as pyramidal neurons, interneurons, and oligodendrocytes based on identification of the most specific markers using sequencing. Repeating bi-clustering on each of the nine major classes identified 47 molecularly distinct subclasses, for example, seven subclasses of pyramidal neurons, 16 subclasses of interneurons, and six subclasses of oligodendrocytes [59]. Another study analyzed 1,679 cells from the primary visual cortex in the adult mouse and identified 49 transcriptomic cell types, such as 23 GABAergic interneurons and 19 glutamatergic neurons [60]. While conventional research has only identified four interneuron subclasses, the single cell sequencing method has classified interneurons into 16 subclasses in the somatosensory cortex and 23 subclasses in the visual cortex, which allows further investigation of the functional complexity of these cells and brain regions [61].

Furthermore, patch-sequencing, which combines the patch-clamp technique with single cell RNA sequencing, allows us to investigate the link between molecularly defined neuronal subtypes and their corresponding electrophysiological and morphological counterparts [31]. Using this approach, 58 cells from layer 1 (L1) of the mouse neocortex have been classified into two cell types: elongated neurogliaform cells (eNGCs) and single bouquet cells (SBCs). A number of the SBCs express genes that are associated with human disease, for example *Npas1* and *Npas3*, have been implicated in autism spectrum disorders. Moreover, a scalable approach to sequence and quantify RNA molecules from isolated neuronal nuclei from a postmortem human brain was developed, generating 3,227 sets of single-neuron data from six distinct regions of the cortex and identifying 16 neuronal subtypes (eight excitatory and eight inhibitory) [62]. In addition, 35,289 single nuclei from the visual cortex, frontal cortex, and the cerebellar hemisphere of a postmortem human brain were analyzed using an improved high-throughput method for single-nucleus droplet-based sequencing (snDrop-seq), providing a more complete brain cell atlas [63].

These reports have demonstrated the advantage of single cell sequencing to identify complex individual cell types in rodent and human brains at a more precise molecular level, which could not be done previously using conventional approaches (Table 1).

#### Retina

The mammalian retina consists of more than 60 distinct cell types, each playing a specific role in processing visual images [64]. Retina neurons were previously defined by their morphology and physiology, and their complexity has been underestimated. Using Drop-seq analysis, about 44,808 mouse retinal cells were analyzed and 39 transcriptionally distinct cell classes identified [9]. Even though amacrine cells account for only about 10% of retina cells, the dataset has revealed that amacrine cells display the most complex subclasses [9]. From another study, retina bipolar cells (BCs) were sorted out from a transgenic mouse line in which GFP is expressed in all BCs. Using Drop-seq, 15 types of BCs were identified and two novel types were discovered according to the molecular diversity of BCs [65, 66]. Thus, single cell RNA sequencing has revealed the unexpected molecular complexity of retina cells that comprise relatively small populations, such as amacrine cells and bipolar cells, and helped further understanding of the function of the retina (Table 1).

#### Hypothalamus

The hypothalamus is capable of sensing light, glucose, and blood osmolarity, and is also one of the brain regions with the highest diversity of neurons [67]. Understanding the cell composition and features of cell type-specific transcription in the hypothalamus is essential to explore the function of this important brain region. One study analyzed 3,131 hypothalamus cells and identified 62 subtypes, such as glutamatergic, dopaminergic, and GABAergic neurons, using single cell RNA sequencing [68]. Another study defined 11 non-neuronal and 34 neuronal cell clusters in the adult mouse hypothalamus [69]. Most of the non-neuronal cell types identified in this study are similar to those found in the cerebral cortex [59]. In contrast, the neuronal cell types are predominantly hypothalamus-specific, indicating that different neuronal components are the basis of different functionalities in different brain regions.

#### Olfactory system

The mammalian olfactory system is capable of detecting and discriminating between a tremendous number of odorants by following the “one neuron–one receptor” rule. Olfactory sensory neurons (OSNs) detect odorants by expressing one olfactory receptor gene (*Olfr*) in each neuron [70]. A study analyzed 178 cells from the main olfactory epithelium of adult and newborn mice using single cell RNA sequencing [71]. Surprisingly, a subset of cells were found to express multiple *Olfrs*, and most of these cells are developmentally immature OSNs [71]. Another study also used single cell RNA sequencing to analyze transcriptomes of single epithelial neurons during development by incorporating cell stage markers in individual transcriptomes which reflect the developmental progression from progenitors to mature OSNs. This study showed that immature OSNs express low levels of multiple *Olfrs*. As development proceeds, this ability declines and mature OSNs typically express high levels of a single *Olfr* [72]. Moreover, immature OSNs are not restricted to expressing *Olfrs* from a single chromosomal region [72]. These studies have revealed a new olfactory development model in which the maturation of OSNs is associated with transition of their expression of low levels of multiple *Olfrs* to high levels of a single *Olfr* (Table 1).

### Exploring human brain development at the single cell level

The human brain is a complex and highly evolving structure. Single cell sequencing has allowed us for the first time to dissect the cell type diversity and pedigree trajectory of the human brain at the molecular level.

#### Neural stem cells and progenitors in the human brain

Radial glia cells (RGCs) are neural stem cells that generate cortical neurons and serve as scaffolds for migrating neurons in the mammalian cortex [58]. RGCs have been classified into apical RGCs (aRGCs) that reside in the ventricular zone (VZ) and basal RGCs (bRGCs) that are located in the outer subventricular zone (OSVZ) in the human brain cortex. Although aRGCs and bRGCs have different morphologies, the diversity of their molecular features is unknown. It has been proposed that bRGCs drive the dramatic cortical expansion observed in gyrified brains, such as the human brain [73, 74]. Single cells were captured from microdissected specimens from the VZ and SVZ of the human cortex at gestational weeks 16–18 (GW16–18) for single cell RNA sequencing [75]. bRGCs were found to preferentially express genes related to extracellular matrix formation, migration, and stemness of neural stem cells, including *TNC*, *PTPRZ1*, *FAM107A*, *HOPX*, and *LIFR* [75]. Gene enrichment analysis identified the selective expression of genes in the LIFR/STAT3 self-renewal pathway in bRGCs, which suggests that expansion of bRGCs in the OSVZ relies on local production of growth factors and activation of the LIFR/STAT3-mediated signaling pathway.

In addition, a method called fixed and recovered intact single cell RNA (FRISCR) has been developed. It enables targeted mRNA purification and single cell transcriptomic profiling of fixed cells without compromising data quality compared with live cells [76]. Using FRISCR, aRGCs have been found to express *ANXA1* and *CRYAB*, while bRGCs express *HOPX*, which further indicates complex lineage relationships in the human brain [76]. Moreover, a recent study analyzed 2,309 single cells from the human fetal prefrontal cortex (PFCs) between gestational week 8 (GW8) and GW26 and classified PFCs into six major cell types, such as neural progenitor cells (NPCs), excitatory neurons, and interneurons [77]. This study has found that bRGCs actively proliferate from GW9 to GW16 and undergo neuronal differentiation from GW16 to GW26 [77]. Interestingly, this report has also revealed that intermediate progenitor cells (IPCs) comprise two types produced at two key time points; one is mainly produced by aRGCs at GW10 and the other is produced by bRGCs at GW16 [77].

#### Modeling human brain development

To study the complexity of human brains, systems for modeling human brain development using human embryonic stem cells (hESCs) and induced pluripotent stem cells (iPSCs) in 2D or 3D culture systems have been developed [78–80]. However, whether these in vitro models can faithfully recapitulate human brain development at the cellular and molecular levels remains unclear.

A recent study analyzed 4,368 progenitor-enriched and neuron-enriched cells, which differentiate from hESCs, after in vitro culturing at multiple points such as day 0 (D0), D12, D26, and D54 using single cell sequencing [81]. Clustering single cell transcriptomic data have identified 41 distinct populations, for example, 14 types of progenitors and 19 types of neurons [81]. Moreover, comparisons with primary mouse and human brain cell expression datasets and creation of lineage trees using the Bayesian algorithm have revealed the importance of Wnt/β-catenin signaling in autonomously controlling cell fate commitment of hESC-derived neural progenitors [81, 82]. In another study, human cerebral organoids were generated from ESCs and iPSCs. After single cell sequencing, single cell transcriptomic data from organoids were analyzed in parallel with molecular markers of primary human fetal cortex [83]. Interestingly, organoid cortical cells have been found to display remarkably similar gene expression profiles to those in the human fetal cortex [83]. Because samples of primary human fetal brains are extremely rare and difficult to collect, the organoid culture system, in combination with single cell sequencing, has provided a valuable vehicle to understand early patterning and regional formation of the human fetal brain and to reveal critical genes that are responsible for early human brain malformation.

## Conclusions

Single cell sequencing is a powerful tool in the fields of stem cell biology and neuroscience. Single cell sequencing allows classification of cell types and subtypes in the CNS at the transcriptome level, which can help to solve the mystery of the heterogeneity and complex function of the nervous system and uncover mechanisms of brain formation at different developmental stages. Single cell sequencing also helps to understand the molecular relationship between disease status and cellular response and reveals specific neuronal cell types in association with different neurological diseases.

Technically, though single cell sequencing provides useful information on gene expression in individual cells, it has relatively low detection efficiencies and requires cells to be removed from their native environment, resulting in the loss of spatial information [84]. Thus, in situ single cell sequencing is a method to overcome these drawbacks [84–86].

Moreover, future development of single cell sequencing should aim to be more refined, high-throughput, economical, and easier to handle in standard research and clinical laboratories. Nevertheless, single cell sequencing has opened a new frontier to classify distinct cell types, identify new biomarkers, and reveal novel mechanisms of CNS development, function, and disorders. Single cell sequencing is becoming a valuable and unique tool in addition to classic morphological methods and conventional cellular and molecular approaches.

## Abbreviations

aRGC: Apical radial glia cell
BC: Bipolar cell
bRGC: Basal radial glia cell
CNS: Central nervous system
CNV: Copy-number variant
DOP-PCR: Degenerate oligonucleotide-primed PCR
eNGC: Elongated neurogliaform cell
FACS: Fluorescence-activated cell sorting
FRISCR: Fixed and recovered intact single cell RNA
GEM: Gel bead in emulsion
GW: Gestational week
hESC: Human embryonic stem cell
IPC: Intermediate progenitor cell
iPSC: Induced pluripotent stem cell
IVT: In vitro transcription
LCM: Laser capture microdissection
LIANTI: Linear amplification via transposon insertion
MALBAC: Multiple annealing and looping base amplification cycles
MDA: Multiple displacement amplification
MMLV: Moloney murine leukemia virus
NGS: Next-generation sequencing
NPCs: Neural progenitor cells
Olfr: Olfactory receptor gene
OSNs: Olfactory sensory neurons
OSVZ: Outer subventricular zone
PCA: Principle component analysis
PFC: Prefrontal cortex cell
RGC: Radial glia cell
SBC: Single bouquet cell
SNES: Single nucleus exome sequencing
SPLiT-seq: Split-pool ligation-based transcriptome sequencing
UMI: Unique molecular identifier
VZ: Ventricular zone
WGA: Whole-genome amplification
WTA: Whole-transcription amplification
